# Identification and potential role of telocytes in human uterine leiomyoma

**DOI:** 10.1186/s40834-016-0022-5

**Published:** 2016-07-20

**Authors:** Essam R. Othman, Dalia A. Elgamal, Abeer M. Refaiy, Ibraheem I. Abdelaal, Asmaa F. Abdel-Mola, Ayman Al-Hendy

**Affiliations:** 1 OB-GYN Department, Assiut University, Assiut, Egypt; 2 Center of Excellence of Stem Cells and Regenerative Medicine, Assiut University, Assiut, 71111 Egypt; 3 Histology Department, Assiut University, Assiut, Egypt; 4 Pathology Department, Assiut University, Assiut, Egypt; 5 OB-GYN Department, Medical College of Georgia MCG, Augusta Univerity, Augusta, GA USA

**Keywords:** Telocytes, Myometrium, Leiomyoma, Stem cells

## Abstract

**Background:**

Telocytes are specialized interstitial tissue cell type. Our aim is to characterize telocytes in human uterine leiomyoma (ULM) and its adjacent myometrium (Myo-F) as well as normal myometrium (Myo-N).

**Methods:**

ULMs and Myo-F tissues were taken from hysterectomy specimens done to treat symptomatic uterine fibroids (*N* = 20). Myo-N is isolated from hysterectomies done on ULM- free uteri for other benign indications (*N* = 15).

Telocytes were detected using immunohistochemistry to detect c-Kit (CD-117), as a surface marker expressed on telocytes, and electron microscopic examination to identify telocytes characteristic ultrastructure. Cellular count and electron microscopic features of telocytes in each of the studied tissues were compared.

**Results:**

Telocytes could be detected in ULMs, Myo-F and Myo-N using c-KIT immunostaining. Electron microscopy confirmed the presence of telocytes in the three types of tissues identifying their characteristic features including small triangular or fusiform cell bodies with extensive cellular prolongations. ULM telocytes showed ultrastructural features suggestive of high cellular activities. Cell counts of ULM telocytes (3.35 ± 0.39) were significantly higher (*P* value = 0.00039) than that of Myo-F (1.39 ± 0.13). Myo-N (2.6 ± 0.36) contained higher telocyte numbers than Myo-F (1.39 ± 0.13), but the difference did not reach statistical significance (*P* value = 0.19).

**Conclusions:**

Telocytes are detected in higher numbers and activity in ULMs than Myo-F or Myo-N. In ULMs, telocytes can work as a hormonal sensors for stem cells, provide scaffold for newly formed myocytes, or control important downstream signaling pathways.

## Background

Uterine leiomyomas (ULMs) are benign, monoclonal neoplasms of the myometrium [1]. They are present in up to 77 % of pathologically examined hysterectomy specimens [2]. Their cumulative incidence at age of 50 years is 80 % for African American women and 70 % for White women [3, 4]. ULMs are a significant cause of pelvic pain, abnormal uterine bleeding, infertility, and pregnancy complications [5]. There is no effective long term medical treatment for women with ULMs [6]. In the US, around 200,000 hysterectomies and 30,000 myomectomies are performed annually to treat such women [7], with annual costs of 4.9 to 34.6 billion USD [8].

The myometrium, which gives origin to ULM, consists of two main cell types; myometrial and interstitial cells. Among interstitial cells, Telocytes have recently been described [9, 10]. Under electron microscope, telocytes have a small, oval, or triangular-shaped body. Their characteristic feature is the presence of very long prolongations called telopodes, usually two to five per cell [11–13]. Telocytes have been described in the stroma of several major organs as heart [14–16], skeletal muscles [17], vessels [18], placenta [19], small intestine [9], and lungs [20]. Telocytes, via direct cell body and with their long branching telopodes, make a 3D network of homocellular or heterocellular contacts [11, 21].

Telocytes may function as a scaffold to define the correct organization of extracellular matrix during tissue repair/renewal [12, 22]. They may also participate in intercellular signaling, immune surveillance and tissue regeneration [21]. Telocytes show waves of depolarization and may participate in spreading the slow waves generated by the pacemaker interstitial cells of Cajal (ICC) in the GIT [23].

In the female genital tract, telocytes have been described in the placenta [19], endometrium [24] and myometrium [10, 25, 26]. However, to the best of our knowledge, this cell type has never been identified in uterine leiomyoma, despite that a considerable interest is currently being given to the potential role of telocytes in pathological conditions of different organ systems [27–29].

## Methods

### Human tissue

Samples of ULMs and Myo-F were taken from women undergoing hysterectomy for the treatment of ULMs (*n* = 20). Hysterectomy patients (*n* = 20) were premenopausal (age range 40–44 years) and in proliferative phase of the menstrual cycle. Fibroids were diagnosed preoperatively using ultrasound and confirmed after surgery with histopathological evaluation. We included other 15 myometrial samples obtained from women undergoing hysterectomy for benign indications other than ULMs (irregular bleeding, chronic pelvic pain and uterine prolapse) as control samples (Myo-N). Women from whom Myo-N were obtained were operated upon in the proliferative phase, and were of comparable age group to the ULMs group (premenopausal). Cycle phase was determined using patient’s menstrual history and histopathology of endometrial samples. All patients gave their informed consent and Assiut Faculty of Medicine Review Board approved the use of human tissues for the study.

### Immunohistochemistry

The presence CD117 protein (c-Kit) was analyzed by immunohistochemical staining using the avidin-biotin immunoperoxidase complex technique.

Immunohistochemistry was performed as manufacturer’s protocol. Tissue sections (4-μm thick) of formalin-fixed, paraffin-embedded specimens were cut. The sections were deparaffinized, rehydrated in graded alcohol, and endogenous peroxidase were blocked by the use of 3 % hydrogen peroxide in methanol for 5 min. Antigen retrieval was done by immersing the slides in citrate buffer and putting them in microwave for 20 min. Samples were then incubated overnight at room temperature with primary antibody for CD117/c-kit (rabbit polyclonal antibody, Thermo scientific, Fremont, CA, cat. no. RB-1518-P0) at a dilution of 1:100. We then used a secondary antibody detection system (Ultravision detection system, Anti-polyvalent, HRP/DAB, Thermo scientific, Fremont, CA, cat. no. TP-015-HD). This was followed by slide developing using 3-3′-diaminobenzidine chromogen and counterstained with Mayer’s hematoxylin. Negative control slides were done by omitting the primary antibody. Sections from gastrointestinal stromal tumor (GIST) were stained as a positive control.

### Evaluation of immunohistchemistry

Stained slides were examined to identify the number of c-KIT positive cells in high power field. For each section 10 non-overlapping randomly selected high power fields were counted and then the average was calculated.

### Electron microscopic examination

Small pieces (1 mm) of leiomyoma and myometrial tissues from patients and controls were excised. Tissue pieces were fixed in 2.5 % glutaraldehyde solution at at 4c for 24 h and washed overnight in several changes of 0.1 mol/l sodium phosphate buffer (pH 7.4) at 4C. Later they were postfixed in 2 % osmium tetraoxide in 0.1 mol/l sodium phosphate buffer at room temperature. The specimens were dehydrated in ascending grades of cold ethanol, and then embedded in epoxy resin. Semi-thin sections (0.5–1-μm-thick) were cut on a Reichert Ultracut ultramicrotome and stained with 1 % (*w/v*) toluidine blue in 1 % (*w/v*) borax for evaluation. Ultrathin sections (70–90 nm) of selected areas of osmicated tissues were cut with a diamond knife, mounted on 200-mesh copper grids and contrasted with 3 % aqueous uranyl acetate for 10 min then in lead citrate for 5 min. The sections were then examined and photographed using a transmission electron microscope JEOL (JEM-100 CXII, Akishima, Tokyo, Japan) and photographed at 80 kV in Assiut University, Electron Microscope Unit.

### Statistical analysis

Average numbers of C-kit positive cells in the three tissue groups (uterine leiomyoma, myometrium adjacent to leiomyom and control myometrium) were compared using Student-*t*-test. Data were expressed as mean ± standard error of the mean (SEM).

## Results

### Light microscopic results

**Fig. 1:**
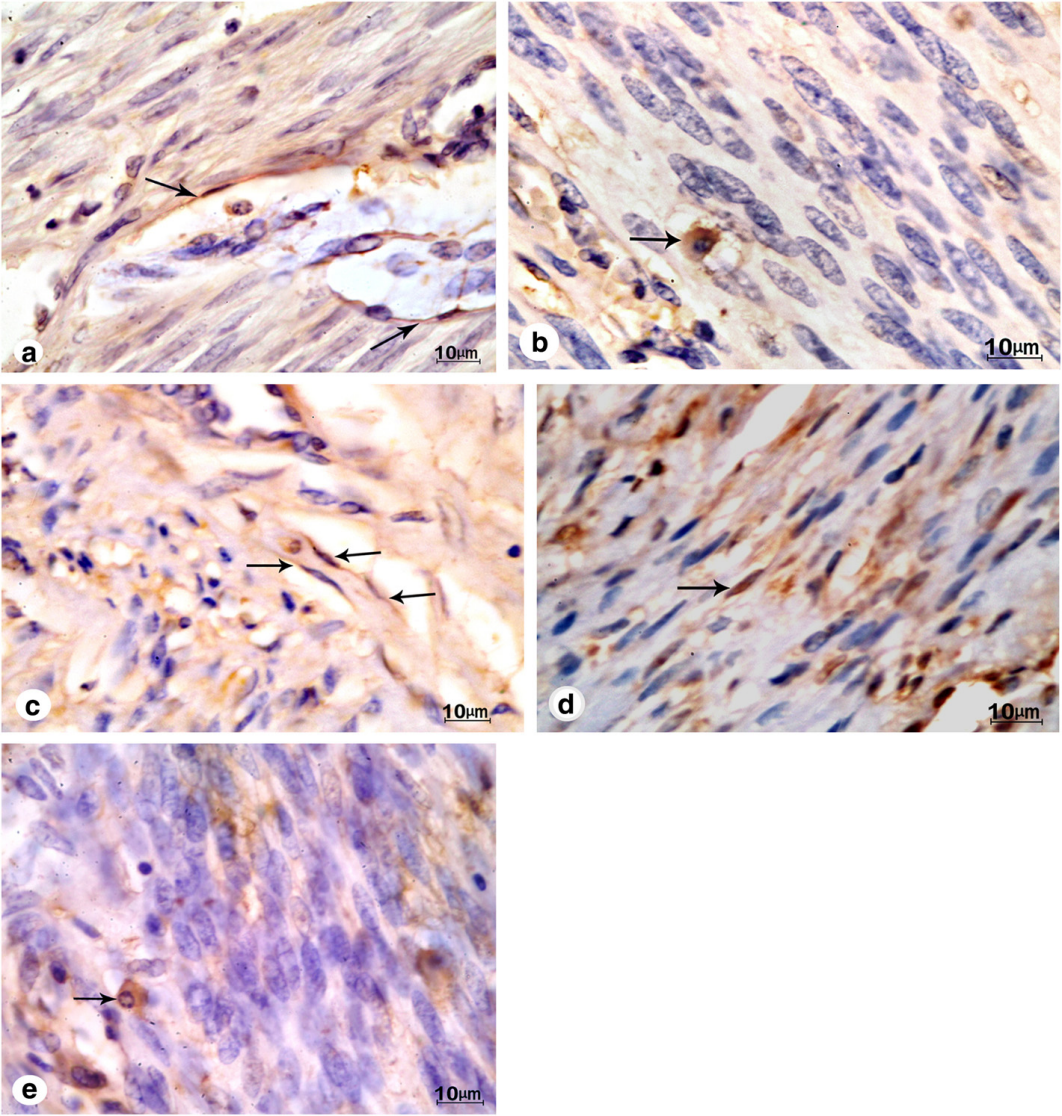
**a** A photomicrograph of a section in the control myometrium showing c-kit positive spindle shape telocytes (*arrow*) among smooth muscle cells. Original magnification ×1000. **b** Another section of the control myometrium showing c-kit-positive mast cell (*arrow*). It is well demarcated by its large rounded nucleus and voluminous cytoplasm. Original magnification ×1000. **c** A photomicrograph of a section in the myometrium adjacent to uterine leiomyoma showing many c-kit positive telocytes with their long extending telopodes extending from the cell body (*arrow*). Notice their intimate relation to blood vessels. Original magnification ×1000. **d** A photomicrograph of a section in the uterine leiomyoma showing c-kit positive telocyte parallel to smooth muscle cells (*arrow*). Original magnification ×1000. **e** Another section in the uterine leiomyoma showing C-kit-positive mast cell with voluminous granular cytoplasm (*black arrow*). Original magnification ×1000

Expression of transmembrane surface receptor c-KIT (CD-117) is characteristic for telocytes in the human myometrium. Few scattered c-kit-positive telocytes were detected among smooth muscle cells of the Myo-N. They appeared as small fusiform cells or cells with scanty cytoplasm and large flat nuclei. Long cytoplasmic processes that extend from the poles of the cell body were identified (telopodes) (Fig. 1a). Mast cells were also immunopositive for c-KIT (CD 117), but can be recognized by their characteristic morphology. They had large rounded nuclei and granular cytoplasm (Fig. 1b). In Myo-F; telocytes were well identified in intimate relation to blood vessels (Fig. 1c). In ULM tissues, the c-KIT positive telocytes as well as mast cells were identified ad found to be located either parallel to myocytes or in relations to blood vessels (Fig. 1d).

### Ultrastructural results

**Fig. 2:**
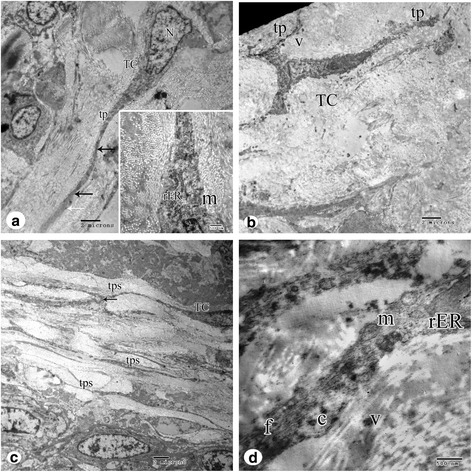
**a** An electron micrograph of a telocyte (*TC*) of the control myometrium showing; pyriform shaped cell body with an oval euchromatic nucleus (*N*), cytoplasm accommodates mitochondria (*m*) and endoplasmic reticulum (*ER*) and thin long processes or telopodes (*tp*) with alternating podomers (*black arrows*) and podoms (*white arrow*). Original magnification ×3600, Inset ×14,000. **b** An electron micrograph of myometrium adjacent to uterine leiomyoma showing a triangular shaped telocyte (*TC*) with numerous shed vesicles (*v*) observed near the telopodes (*tp*). Original magnification is ×3600. **c** An electron micrograph of uterine leiomyoma showing multiple telocytes (*TC*) with their long telopodes (*tps*) in a labyrinthine configuration among muscle cells. Intercellular junction exists between telopodes of different telocytes (*black arrows*). Original magnification is ×2900. **d** Higher magnification of telopodes show dilated rough endoplasmic reticulum cisternae (*rER*), mitochondria (*m*), microfilaments (*f*), caveolae (*c*) and shed vesicles (*v*). Original magnification ×19,000

Telocytes are distinguished in the present work in the interstitial space between smooth muscle cells. Ultrastructural examination of Myo-N showed that a telocyte have pyriform shape cell body with cellular prolongations extending from either sides of the cell called telopodes. The cell body contains an euchromatic nucleus with clusters of peripheral heterochromatin clumps. The nucleus is surrounded by a small amount of cytoplasm rich in mitochondria and rough endoplasmic reticulum. Telopodes exhibit the presence of podomeres (extremely thin segments) and podoms (dilated portions) (Fig. 2a and inset).

Uterine telocytes detected in Myo-F lie around smooth muscle cells or in the viscinity of blood capillaries (Fig. 2b). Their nuclei showed well defined nucleoli. Telopodes run in labrynthine manner and shed vesicles were abundant (Fig. 2b).

Ultrathin sections of ULM showed telocytes with extremely long telopodes wich achieved contact between different telocytes. Their nuclei were euchromatic,. The cytoplasm was rich in mitochondria, dilated rough endoplasmic reticulum cisternae, microfilaments, caveoli, and shed vesicles (Fig. 2c and d).

### Cell counting

**Fig. 3:**
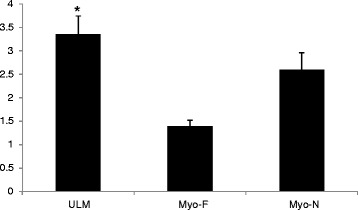
Average count of c-Kit positive telocytes per high power field in uterine leiomyoma (ULM), myometrium adjacent to leiomyoma (Myo-F), and normal myometrium (Myo-N). * denotes significantly higher cell number as compared to myometrium adjacent to leiomyoma (Myo-F)

Under light microscope, c-KIT positive telocytes, with their characteristic morphology, were counted in 10 high power fields and averaged for each of ULM, Myo-F, and Myo-N. The highest telocyte cell numbers were identified in ULM tissues (3.35 ± 0.39), whereas Myo-F showed the fewest number of telocytes (1.39 ± 0.13). Myo-N exhibited an intermediate telocyte cell number (2.6 ± 0.36). On comparing the three studied tissue groups, significantly higher telocyte numbers were detected in ULM as compared to Myo-F. No significant difference was found between Myo-F and Myo-N, or between ULM and Myo-N (Fig. 3).

## Discussion

ULMs, despite being the most common gynecologic tumors, and associated with significant reproductive morbidity, their exact etiology is not understood.

In the present study, we have characterized, for the first time to the best of our knowledge, a specialized interstitial cell type; telocytes; in ULM and Myo-F. Our results show that as compared to Myo-F or Myo-N, ULMs contain higher number of telocytes with ultrastructural features suggestive of increased cellular activity including loss of the heterochromation clumps, extremely long telopods, large numbers of mitochondria, dilated rough endoplasmic reticulum cisternae, and clusters of shed vesicle.

Every effort has been made to correctly identify telocytes in our study. Positive immunostaining to c-Kit (CD 117) was employed as a marker to recognize telocytes [10, 30]. Mast cells, which are also c-KIT positive, were easily distinguished as large round or oval cells with abundant granular cytoplasm and large round nuclei [10, 31]. Moreover, strict ultrastructural criteria were applied to define telocytes under electron microscope. These included: 1) small cell body (9–15 μm), with scanty cytoplasm, 2) spindle or triangular shape, 3) nucleus with moderately dense chromatin, and, 4) most characteristically, the presence of telopodes, identified as very long cytoplasmic processes (2–5 per cell) with a branching pattern and a “bead on string” appearance [9, 10].

In agreement with our results are the findings of previous studies in which telocytes have been identified in the human myometrium [10, 30, 32–34]. The function of telocytes in the myometrium is still controversial. Being morphologically similar to Interstitial Cells of Cajal (ICC) of the GIT that regulate gut motility, uterine telocytes are assumed to be the pace maker controlling myometrial contractions. Supporting this concept is electrophysiological data showing that myometrial telocytes of the non pregnant uterus exhibit hyperpolarizing chloride inward current with calcium dependence indicating a role in myometrial excitability [10, 26, 35]. Telocytes are also involved in intercellular signaling throughout the myometrium and involved in the process of myometrial regeneration [10, 36]. As telocytes communicate with immune cells within the myometrium, these specialized cells may play a role in the immune tolerance to pregnancy [37].

Although our study lacked functional experiments, but our observation of increased telocyte cell number and activity in ULM point out an active role of these cells in ULM development and/or growth.

The cellular origin of ULM is not exactly known. Early genetic studies have indicated that ULMs are monoclonal in origin [38]. It is reported that mouse and human myometrium contain multipotent stem cells that are responsible for tissue regeneration [39, 40]. Similarly, uterine leiomyomas were shown to contain a side population of cells that show phenotypic and functional characteristics of fibroid stem cells [41], which are believed to give origin to fibroid tumors under influence of steroid hormones [42].

To be accepted as cells of origin of such highly steroid dependent tumors, fibroid stem cells should have a mechanism to respond to estrogen and progesterone. However, the side population of fibroid stem cells contains very little amounts of steroid hormone receptors [41, 42]. It is postulated that estrogen and progesterone work on the nearby steroid hormone receptor positive mature myometrial and fibroid cells releasing paracrine factors that stimulate the fibroid stem cells to mediate tumor growth [1]. Telocytes can fit nicely into that model. They have been shown to express estrogen and progesterone receptors in their cell bodies and in cellular processes [43, 44]. Moreover, they keep an extensive network of intercellular communication within the myometrium, and as our findings indicate, within the ULM as well. Based on our findings and those of others, we can postulate that telocytes may work as hormonal sensors for uterine leiomyoma stem cells [44]. Telocytes can respond to estrogen and progesterone hormones through their expressed steroid hormone receptors, and relay their signals to the leiomyoma stem cell through their extensively branching processes.

Telocytes work as part of stem cell niche to form stem cell/telocyte tandem. In leiomyoma, their interstitial network of telopodes can build a dynamic scaffold surrounding the stem cells, which helps to guide the newly formed leiomyoma cells to form a coherent 3D architecture. Such a role of telocytes in nursing stem cells has been shown in cardiac [45, 46], and skeletal muscles [47, 48].

c-KIT (CD 117) expressed by telocytes is a member of type III trans-membrane receptor tyrosine kinase family. Binding of the c-KIT receptor to its ligand, stem cell factor (SCF), results in receptor dimerization and kinase activation. This leads to subsequent activation of two important molecular pathways: the mitogen-activated protein (MAP) kinase pathway (RAF, MEK and ERK), and phosphoinositide 3-kinase (PI3K) pathway (AKT, mammalian target of rapamycin (mTOR), S6 kinase). In addition, signal transducer and activator of transcription 3 (STAT3) is activated. The collective impact favors an increase in cell metabolism, cell cycle progression, and a decreased sensitivity to apoptosis [49].

The ERK signaling pathway, (a downstream of MAP kinase activation) has been shown to be a mediator of ULM growth [50]. In addition, mTOR signaling, (a downstream of PI3K activation), is activated in high frequency in ULM [51, 52]. The role of STAT3 in ULM was elucidated in a study in which tyrosine kinase inhibitor reduced leiomyoma cell proliferation in vitro mediated through phosphorylated STAT3 inhibition [53].

Taken together, it appears that c-KIT receptor activation can stimulate important intracellular pathways that have been implicated in ULM pathogenesis.

Of interest in our study is the finding that Myo-F contained fewer telocytes than the Myo-N, although the difference did not reach statistical significance. In the gastrointestinal tract, disorders characterized by excessive fibrosis, such as ulcerative colitis, Crohn’s disease, are associated with marked reduction in number and/or ultrastructural damage to the affected tissue telocytes [29, 54, 55]. This contributes to the loss of function and dysmotility encountered in the affected structures such as colon or stomach [54]. A similar concept can be extrapolated to the fibroid uterus, where excessive extracellular matrix deposition in ULM is associated with reduction in telocytes number in Myo-F. This can lead to disturbed tissue homeostasis and abnormal contraction waves in the uterus which can be associated with infertility or dysmenorrhea observed in ULM patients.

Alternatively, as telocytes show some degree of motility [13], the reduced number of telocytes in Myo-F compared to Myo-N, may be explained on the basis of migration and recruitment of myometrial telocytes to the region of the ULM.

Further supporting our data on ULMs are previous studies implicating telocytes in the pathogenesis of cancer [56]. Telocytes had potential functions in self-assembly of normal breast stromal, and breast cancer cells to self- assembly breast cancer tissue in vitro. Telocyte-like cells closely communicated with breast cancer cells as well as other stromal cells, and might serve as a bridge that directly linked the adjacent cells [57]. In the skin, and in comparison to telocytes from normal dermis, telocytes from basal and squamous cell carcinoma of the skin showed limitation in their heterocellular junctions. This suggests a possible involvement in induction of cell-cell communication into the tumor [58].

c-KIT expressed by telocytes is amenable for pharmacological manipulation. Imatinib, a 2-phenyl amino pyrimidine derivative, is a tyrosine kinase inhibitor with activity against c-KIT. It works by binding close to the ATP binding site on the tyrosie kinase, locking it in a closed conformation. This process ultimately results in “switching-off” the downstream signaling pathways [59], some of which are essentially important in uterine ULM growth. Imatinib is used as a molecularly targeted therapy for the treatment of cancers such as chronic myeloid leukemia or gastrointestinal stromal tumors (GIST). The drug is given orally and generally well tolerated, with serious side effects rarely happening [60].

## Conclusions

In conclusion, telocytes are detected in higher numbers and activity in ULM than Myo-F or Myo-N. In ULM, telocytes can form a tandem with stem cells working as a hormonal sensors and/or nursing stem cells. c-KIT expressed by telocytes controls downstream signaling pathways essential for ULM growth. c-KIT receptors on human ULMs can be blocked by tyosine kinase inhibitors such as imatinib. This may represent a therapeutic option for human ULMs.
